# Measuring T-Cell Responses against SARS-CoV-2 Is of Utility for Disease and Vaccination Management

**DOI:** 10.3390/jcm11175103

**Published:** 2022-08-30

**Authors:** Guillem Safont, Irene Latorre, Raquel Villar-Hernández, Zoran Stojanovic, Alicia Marín, Cristina Pérez-Cano, Alicia Lacoma, Bárbara Molina-Moya, Alan Jhunior Solis, Fernando Arméstar, Joan Matllo, Sergio Díaz-Fernández, Arnau Cendón, Liliya Sokalchuk, Guillermo Tolosa, Irma Casas, Antoni Rosell, José Domínguez

**Affiliations:** 1 Institut d’Investigació Germans Trias i Pujol, 08916 Badalona, Spain; 2 CIBER de Enfermedades Respiratorias, Instituto de Salud Carlos III, 28029 Madrid, Spain; 3 Universitat Autònoma de Barcelona, 08193 Bellaterra, Spain; 4 Pulmonology Department, Hospital Universitari Germans Trias i Pujol, 08916 Badalona, Spain; 5 Basic Unit for the Prevention of Occupational Risks (UBP), Hospital Universitari Germans Trias i Pujol, 08916 Badalona, Spain; 6 Intensive Care Medicine Department, Hospital Universitari Germans Trias i Pujol, 08916 Badalona, Spain; 7 Diagnostic and Research in Immunodeficiencies Jeffrey Modell Center, Cytometry and Cellular Culture Area, La Frontera University, Temuco 01145, Chile; 8 Preventive Medicine Department, Hospital Universitari Germans Trias i Pujol, 08916 Badalona, Spain

**Keywords:** SARS-CoV-2, T-cell response, humoral response, IFN-γ, vaccination, ELISPOT

## Abstract

The measurement of specific T-cell responses can be a useful tool for COVID-19 diagnostics and clinical management. In this study, we evaluated the IFN-γ T-cell response against the main SARS-CoV-2 antigens (spike, nucleocapsid and membrane) in acute and convalescent individuals classified according to severity, and in vaccinated and unvaccinated controls. IgG against spike and nucleocapsid were also measured. Spike antigen triggered the highest number of T-cell responses. Acute patients showed a low percentage of positive responses when compared to convalescent (71.6% vs. 91.7%, respectively), but increased during hospitalization and with severity. Some convalescent patients showed an IFN-γ T-cell response more than 200 days after diagnosis. Only half of the vaccinated individuals displayed an IFN-γ T-cell response after the second dose. IgG response was found in a higher percentage of individuals compared to IFN-γ T-cell responses, and moderate correlations between both responses were seen. However, in some acute COVID-19 patients specific T-cell response was detected, but not IgG production. We found that the chances of an IFN-γ T-cell response against SARS-CoV-2 is low during acute phase, but may increase over time, and that only half of the vaccinated individuals had an IFN-γ T-cell response after the second dose.

## 1. Introduction

The Severe Acute Respiratory Syndrome Coronavirus 2 (SARS-CoV-2) has infected a total of 552.5 million individuals causing the death of 6.35 million since its appearance in December 2019 [1]. Considered a pandemic since March 2020 after a large global outbreak, it is a major public health concern, filling ICUs and neglecting the control of other diseases [2,3,4]. Whilst most people experience an asymptomatic or a mild SARS-CoV-2 infection, others can present a severe condition usually associated with some specific comorbidities. The severe condition is associated with pneumonia, involving chest pain and hemoptysis, but can also cause organ failure, which can lead to severe sequelae, and even death [5].

Currently, the main available vaccines against SARS-CoV-2 are based on the spike protein of the virus. This glycoprotein has a high antigenicity and is significantly immunogenic when activating the adaptive immune response [6]. At present, the principal vaccines administered against the pathogen are the following: mRNA-1273 (Spikevax, ModernaTX, Inc., Cambridge, MA, USA) and BNT162b2 (COMIRNATY, Pfizer, Inc., and BioNtech, New York, NY, USA), both based on mRNA vaccine design; and ChAdOx1-S (Oxford/AstraZeneca, UK) based on carrier vaccine design. Two doses of these vaccines are recommended, and a third dose is also administered as a booster. Recently, a second booster has been recommended for immunocompromised individuals with a suboptimal immune response to an earlier vaccination [7]. Currently, 67.4% of the world population has received at least one dose of a COVID-19 vaccine, and 12.46 billion doses have been administered globally, contributing to a decrease in the severity and the mortality associated with the pathogen [8].

Humoral, and cellular adaptive responses are fundamental for the virus elimination, the infection resolution, and the protection against reinfection [9,10]. Looking closer at the adaptive response, specific antibodies seem to decline faster than the cellular response. In fact, individuals infected with SARS-CoV, did not show any specific antibody response within two to three years after infection, however, SARS-CoV-specific memory T-cells were detected even after 11 years [11]. Nevertheless, the cellular response against the virus and its mechanisms are far from being completely characterized, so studying it is substantial for a better understanding of the immune response against the virus, the pathogenicity associated, and the long-term immunity that SARS-CoV-2-specific T-cells could be conferring. In addition, the role that vaccination plays in triggering specific T-cell responses against the virus must not be overlooked, as analyzing it can contribute to determine the robustness and the durability of protection [12,13].

In this study, we assessed the cellular immune response by detecting the IFN-γ secreting T-cells after specific stimulation with different SARS-CoV-2 antigens during acute disease and convalescence, and after vaccination. In addition, the IgG and IgM humoral immunity were analyzed and compared with the specific T-cell response.

## 2. Materials and Methods

### 2.1. Study Groups and Clinical Definitions

Blood sample collection took place in the Hospital Universitari Germans Trias i Pujol from July 2020 to November 2021. A signed written consent form was obtained from all subjects included in the study after being informed about the project. The study was approved by the Ethics Committee of the Hospital Germans Trias i Pujol (PI-20-117).

In this study, patients were classified according to disease severity following the advice of the Pulmonology Department COVID-19 Commission of the Hospital Universitari Germans Trias i Pujol. Severity degrees were defined as asymptomatic, mild, moderate, and severe. In our study, patients classified as asymptomatic were health care workers who had a positive SARS-CoV-2 PCR in the context of work setting routine screenings and had no symptoms during the course of infection. Patients were defined as having mild infection if they did not require oxygen support or were only in need of nasal prongs, independently if they were hospitalized or not. Moderate infection was reported in patients admitted to respiratory semi-critical care unit requiring non-invasive ventilation, and severe infection in those hospitalized in intensive care unit (ICU) requiring invasive mechanical ventilation.

A total of 259 samples from 230 individuals were obtained and classified as follows:

1. One hundred twenty-eight samples from controls who were health care workers. selected based on having no prior/present positive SARS-CoV-2 PCR and/or rapid antigen test, and/or having no detectable IgG or IgM plasma antibodies at the moment of inclusion. They were grouped according to SARS-CoV-2 vaccination status as follows: (a) unvaccinated individuals (*n* = 80), and (b) vaccinated individuals with Pfizer (*n* = 47) or Moderna (*n* = 1). Days between sampling and the first/second dose administration were recorded.
2. Seventy-one samples from patients with asymptomatic (*n* = 6), mild (*n* = 3), moderate (*n* = 33), and severe (*n* = 29) COVID-19 disease enrolled during the acute phase of the disease. All patients had a reported positive SARS-CoV-2 PCR and/or rapid antigen test. Inside this group, 19 patients with moderate or severe COVID-19 were followed-up (having two or more consecutive samples, being the overall number of samples 48) during days 0, 2, 7, 28 or discharge after admission into semi-critical or ICU.
3. Sixty samples from individuals recruited during the convalescence phase after mild (*n* = 22), moderate (*n* = 18), or severe disease (*n* = 20). All of them had a record of the number of days between sampling and COVID-19 diagnosis with a positive test.

Overall, a total of 322 samples were collected for the study, although 63 were excluded for not having enough cell counts for T-cell studies. Descriptive demographic and clinical data from individuals included in the analysis have been summarized in Table 1. Data concerning intrinsic information of the samples such as time since diagnosis and vaccination, or lymphopenia at sampling have been included in Supplementary Table S1.

### 2.2. Peripheral Blood Mononuclear Cells (PBMCs) Isolation and Cryopreservation

A total of 16 mL of blood was collected from each patient in CPT tubes (Becton Dickinson Diagnostics, Franklin Lakes, NJ, USA). Next, PBMCs were isolated from blood by density gradient centrifugation. Afterwards, they were washed twice with RPMI (Biowest, Nuaillé, France) supplemented with 10% fetal bovine serum (FBS; Biowest, Nuaillé, France) and finally counted using trypan blue in an inverted microscope. For cryopreservation, cells were suspended in 10% DMSO FBS (Sigma-Aldrich, Saint Louis, MO, USA), transferred into cryovials, and then stored at −80 °C in a cold Nalgene Mr. Frosty Cryo 1 °C Freezing Container (ThermoFisher, Waltham, MA, USA). Cells were then transferred to liquid nitrogen within a week.

### 2.3. ELISPOT Assay for IFN-γ T-Cell Response Detection

The T-cell response from each sample was evaluated by means of an ELISPOT assay (T-SPOT Discovery SARS-CoV-2, Oxford Immunotec, Abingdon, UK). The cells were thawed, and their concentration was adjusted to 2.5 × 10^6^ cells/mL. Next, a total of 250,000 cells per well were stimulated overnight (16–20 h) at 37 °C, 5% CO_2_ with: specific SARS-CoV-2 (a) spike antigen, (b) nucleocapsid (NCP) antigen, (c) membrane antigen, (d) antigens with homology regions of endemic human coronaviruses, (e) AIM-V medium (negative control), and (f) phytohemagglutinin (mitogen; positive control). Peptides from the assay covered the most immunogenic regions of the virus genome, spanning the full length of those proteins, which allowed the study of the extent of T-cell immunity and guaranteed the effect of point mutations was minimized [14]. As detailed by the manufacturer, following the incubation, the IFN-γ released was revealed with a detection antibody and a substrate that showed the IFN-γ secreting cells as spot-forming cells (SFCs). The SFCs were counted for each of the antigens using an automated plate reader (Autoimmun Diagnostika GmbH, Straßberg, Germany) and checked by the naked eye.

The test scored as positive when the final SFCs count was more than 7, even when the sample was unresponsive for the positive control. A borderline result was considered between 5–7 SFCs (both included). SFCs counted in the negative control were always subtracted from the SFCs counted for every specific antigen (antigen SFCs—negative control SFCs). Samples with less than 20 SFCs in the positive control and/or more than 10 SFCs in the negative control were considered indeterminate. The number of reactive T-cells (SFCs) for each of the patient groups enrolled in the study was also analyzed to investigate the quantity of the IFN-γ response.

### 2.4. ELISA for Humoral Response Detection

The IgG concentration against spike was quantified with the QuantiVac ELISA kit according to the manufacturer’s instructions (Euroimmun, Lübek, Germany). Six calibrators at different known concentrations were used to perform a calibration curve. Binding IgG units against the antigen [BAU/mL] were calculated through extrapolation to the curve.

The IgG and IgM against NCP were evaluated with the semi-quantitative Anti-SARS-CoV-2 (IgG) and Anti-SARS-CoV-2 (IgM) ELISA kits (Euroimmun, Lübek, Germany) according to the insert instructions. A ratio was performed between the sample’s and calibrator’s absorbance. Cut-off values for positive, negative, and borderline values were provided by the manufacturer for each test.

### 2.5. Statistical Analysis

The results comparing the IFN-γ response between groups were performed using the two-tailed Mann-Whitney U-test for unpaired comparisons. The differences were considered statistically significant when a *p*-value was <0.05. The correlations were calculated using the two-tailed non-parametric Spearman test. The statistical analyses together with graphical representations were carried out using GraphPad Prism version 8 (GraphPad Software, Inc., San Diego, CA, USA).

## 3. Results

### 3.1. Positivity Rate between Patient Groups

Looking at the overall T-cell response results for specific antigens, the one that triggered more positive responses was spike (46.8% (118/252)). NCP and membrane antigens elicited a positive response in 30.9% (77/249) and 29.3% (73/249) of the samples, respectively (Table 2). In addition, the antigens with homology regions of endemic (human) coronaviruses also induced a response in 25.9% (65/251) of the samples (Supplementary Table S2). In 7 samples the results of the 3 antigens were considered indeterminate due to inadequate responses in the negative or positive controls. These indeterminate samples corresponded to 3 unvaccinated controls and 4 acute patients (1 moderate and 3 severe cases). In addition, 2 samples presented less than 20 SFCs in the positive control; however, as they had a response against the spike and/or the membrane antigens, they were considered positive only for that particular antigen and indeterminate for the others.

As shown in Table 2, the specific response against spike antigen was present in 50% (24/48) of the vaccinated controls. The positivity rate in unvaccinated controls was low (3.9% (3/77) for spike, 1.3% (1/77) for NCP and 0% for membrane). The positive results against NCP and membrane in vaccinated individuals, and the positive results against the three antigens in control unvaccinated individuals, could be due to asymptomatic and/or non-reported SARS-CoV-2 infections. In the acute disease patient’s group, the percentage of positive results against spike, NCP, and membrane was 56.7% (38/67), 48.4% (31/64), and 32.8% (21/64), respectively. In addition, acute severe patients showed a lower percentage of positive results against spike (50% (13/26)) than moderate ones (68.8% (22/32)). In convalescent patients, a total of 91.7% (55/60) of the samples were responsive against at least one SARS-CoV-2 antigen, being the spike and membrane the ones with the highest positivity percentage (88.3% (53/60) and 81.7% (49/60), respectively). Moreover, positivity percentages against any of the antigens among convalescent patients increased according to the severity during the acute phase of the disease.

Regarding the 19 patients with follow-up, positivity percentage against spike was the highest (54.2% (26/48)), increasing according to days of hospitalization (Supplementary Table S3). When analyzing SFCs in these monitored patients, high inter-individual variability was observed although most responses tended to increase. No association was found between the disease outcome (mortality vs. non-mortality) and the T-cell response detected during the days admitted into semi-criticals or ICU (Supplementary Figure S1).

### 3.2. Quantitative IFN-γ Response against SARS-CoV-2 Antigens

**Figure 1:**
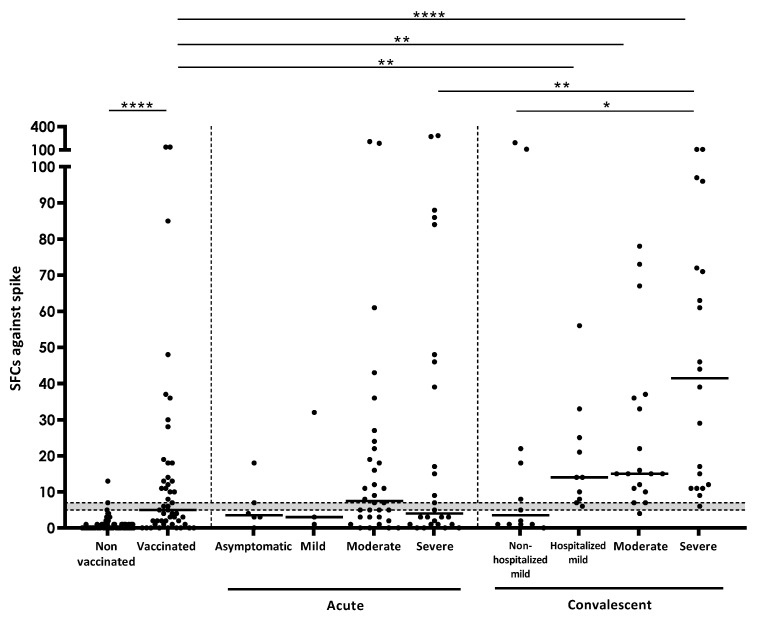
Number of SFCs after stimulation with spike in the different study groups. Horizontal lines represent medians. Grey area shows borderline results. Differences between conditions were calculated using the two-tailed Mann-Whitney U test. *p* is considered significant when <0.05 (* <0.05, ** <0.01, and **** <0.0001).

In addition to positivity rates described in the previous section, the number of responding T-cells after specific stimulation was also investigated. When the quantity of this response (measured as SFCs) against spike was analyzed in acute COVID-19 patients, a reduced IFN-γ response was observed in asymptomatic and mild subgroups. Moderate acute COVID-19 patients displayed a greater response than severe acute COVID-19 patients, however, there was no statistical significance when comparing the four subgroups (Figure 1). Moreover, the number of lymphocytes/μL in acute COVID-19 patients moderately correlated with the IFN-γ T-cell and IgG response against spike (SR = 0.318; *p* = 0.019 for T-cells; SR = 0.553; *p* = 0.009 for IgG; Supplementary Figure S2a,b). Regarding convalescent patients, the number of SFCs against spike increased according to disease severity in the acute phase, and it was significantly higher in patients who passed severe COVID-19 compared to mild COVID-19 patients who were not hospitalized (*p* < 0.05). Moreover, mild, moderate, and severe COVID-19 convalescent subgroups had a significantly higher response than vaccinated controls (*p* < 0.01 when comparing mild or moderate; *p* < 0.0001 in severe) (Figure 1). No more significant differences were found between groups.

Regarding NCP and membrane, none of the patients with acute COVID-19 showed a median response above the positivity threshold. Although not significant, convalescent subgroups had a higher average response than acute and control groups for both antigens. In convalescent individuals, the response against the membrane antigen was significantly lower in mild non-hospitalized individuals than in the rest of the groups (*p* < 0.0001) (Supplementary Figure S3a,b).

### 3.3. IFN-γ Response According to Days after Vaccination and after COVID-19 Diagnosis

**Figure 2:**
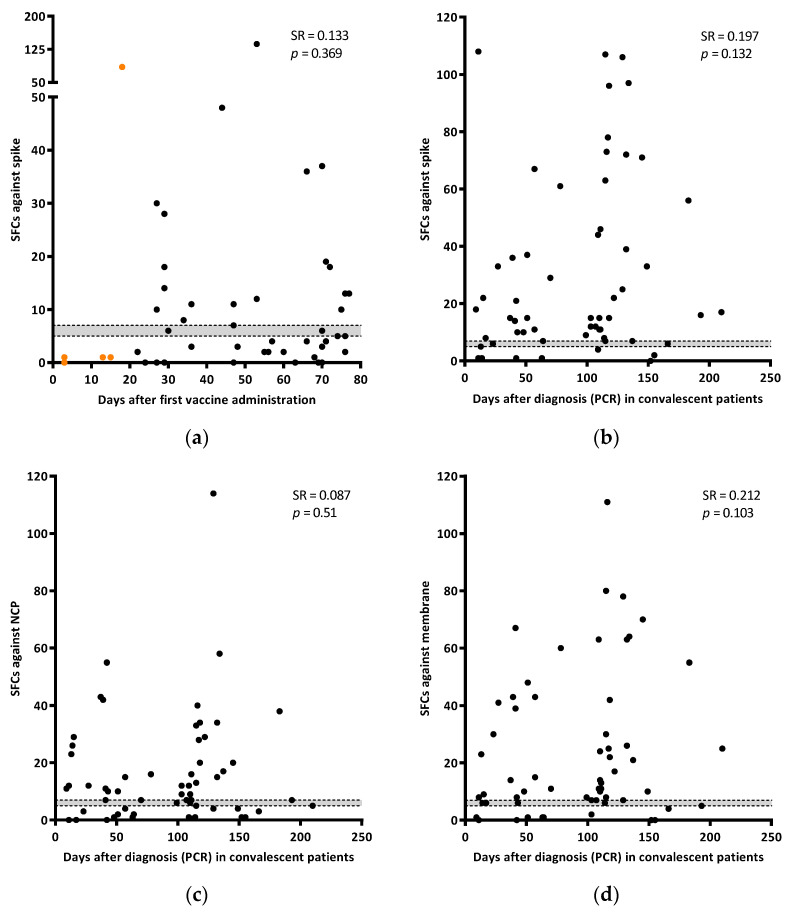
Correlation of the IFN-γ response against spike (SFCs) with days after the first dose of the vaccine administration (**a**). Orange dots are samples from individuals with only one dose. Correlation of the IFN-γ response (SFCs) in spike (**b**), NCP (**c**), and membrane (**d**) with days after diagnosis (PCR) in convalescent patients. Grey area shows borderline results. Correlations were calculated using the two-tailed non-parametric Spearman test.

The days between sampling and the first/second vaccine dose administration were documented. As shown in Figure 2a, there was not a significant correlation (SR = 0.133; *p* = 0.369) between the IFN-γ response and time after the first vaccine dose. A total of 46.5% (20/43) of individuals did not show response against spike after receiving the two vaccine doses (between 21–77 days after the first dose administration) (Figure 2a).

The T-cell response against any of the three SARS-CoV-2 specific antigens tested was analyzed in convalescent patients since diagnosis. Although responses tended to increase along time, no correlation was found between the antigens evaluated and time (SR = 0.197; *p* = 0.132 for spike, SR = 0.087; *p* = 0.51 for NCP; and SR = 0.212; *p* = 0.103 for membrane) (Figure 2b–d).

### 3.4. T-Cell IFN-γ Production and Humoral Responses

**Figure 3:**
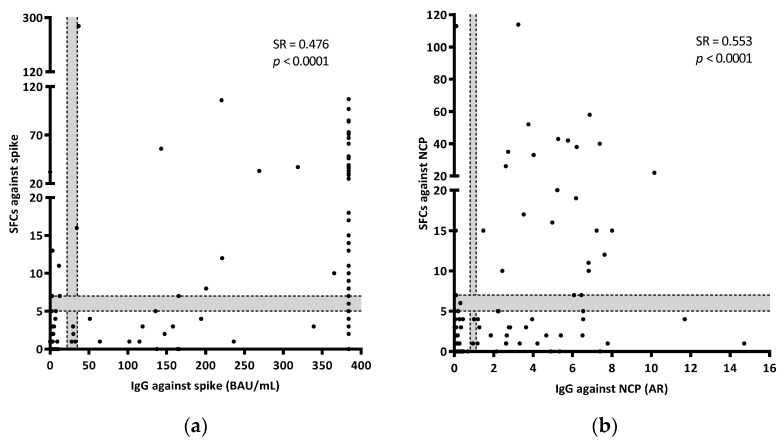
Correlations between the IFN-γ T-cell (SFCs) and IgG responses against spike (**a**) and NCP (**b**). AR refers to Absorbance Ratio (sample Abs/calibrator Abs). BAU refers to Binding Antibody Units. Grey areas show borderline results. Correlations were calculated using the two-tailed non-parametric Spearman test. In Figure 3a, results out of the calibration curve (equal or over 384 BAU/mL) were excluded from the Spearman test.

In a subgroup of 159 samples, it was possible to evaluate IgGs against spike and NCP, as well as IgMs against NCP. Four of them had indeterminate results for the T-cell test. Overall, the number of positive results obtained by detecting IgG was higher than that obtained when detecting the IFN-γ producing T-cells (55.5% (86/155) IgG vs. 40.6% (63/155) T-cell against spike; 35.7% (55/154) IgG vs. 20.8% (32/154) T-cell against NCP) (Table 3). In addition, levels of IgG specific for spike and NCP antigens significantly correlated with SFCs. For both cases, there was a moderate correlation between antibody and cellular responses (for IgG spike SR = 0.476, *p* = <0.0001; for IgG NCP SR = 0.553, *p* = <0.0001) (Figure 3a,b). Unvaccinated controls showed low percentages of positive responses against both spike (9.7% (6/62) and 4.8% (3/62) for IgG and T-cell responses, respectively) and NCP (6.5% (4/62) and 1.6% (1/62) for IgG and T-cell responses, respectively). In the vaccinated group, the number of positive results when evaluating spike was higher when detecting the humoral response than when detecting T-cell responses (90% (27/30) and 46.7% (14/30), respectively). Considering patients with acute COVID-19, IgGs against spike were present in 67.6% (23/34) of the samples compared to 52.9% (18/34) when detecting a T-cell response against the same antigen. Similarly, 69.7% (23/33) and 38% (12/33) of the samples were positive for IgG and T-cell responses against NCP, respectively. The number of positive results were comparable between IgG and cellular responses in convalescent patients for spike (100% (29/29) and 95.6% (28/29), respectively), but not for NCP (95.6% (28/29) and 69% (20/29), respectively) (Table 3). Regarding IgM against NCP, patients with acute disease showed an amount of positive results, similar to that obtained by studying T-cell responses (27.3% (9/33) for IgM and 38% (12/33) for T-cells). A lower rate of positive IgM results was observed in controls and convalescent participants (no response in controls (0/92), and 3.6% (1/29) in convalescents; data not shown).

Thirty-three samples (21.3%) showed discrepancies when comparing IgG response against spike and/or NCP and T-cell response against any SARS-CoV-2 antigen. In this case, 27 of the 33 (81.8%) discrepancies were as a result of having IgGs but no T-cell response, and the opposite happened with the other 6 samples. Most of the samples with IgGs but no T-cell response were from vaccinated controls (51.9% (14/27)). In this case, 11 discrepancies were found in acute patients, being 4 of them (36.4%) positive for T-cell but not for IgG. Convalescent individuals did not show any discrepancies (Supplementary Table S4).

## 4. Discussion

The study and monitoring of the specific cellular and humoral responses against SARS-CoV-2 are of foremost importance for a better understanding of immunity during the acute phase of the disease and the long-lasting immunity after infection and/or vaccination, and probably in the long-COVID-19 syndrome. In this study we assessed the IFN-γ T-cell immune response against specific SARS-CoV-2 antigens and compared it with the humoral response in patients undergoing acute disease, during convalescence, and in unvaccinated and vaccinated individuals. Our results indicate that the IFN-γ T-cell response against SARS-CoV-2 is low during the acute phase of the disease but can increase over time as seen in this study in the case of convalescent individuals. Moreover, the overall cellular immune response triggered by vaccination was low, as around half of the vaccinated individuals did not show a response after the second dose administration, but humoral response is detected in the majority of cases. Finally, IgG levels correlated with the number of IFN-γ releasing T-cells.

In our study, a low IFN-γ T-cell response was observed in individuals undergoing the acute phase of the disease, particularly in severe patients. One plausible reason that can explain this is the inclusion of patients who have not yet developed an adaptive response in the initial phases of the disease. The IFN-γ release is essential for fighting viral infections, however, it is still not yet clear the strength of T-cell immune response during severe illness, as controversial data indicate that severe COVID-19 patients can have an insufficient but also an excessive response. Some manuscripts have reported that lymphopenia and anergy in severe acute patients may be a reason for that lack of cellular response [15,16,17,18]. Our results are consistent with these previous studies, indicating that lymphopenia and lower IFN-γ T-cell responses could correlate with disease severity [15,17,18,19]. In fact, the majority of samples initially excluded from the study with not enough cell counts were samples from moderate or severe COVID-19 patients (68.3%). Although, humoral response was seen in a higher number of acute COVID-19 patients than the T-cell response, in some samples no humoral response was seen while an IFN-γ T-cell response was detected. That strengthens the fact that the study of the T-cell responses could be important in COVID-19 management.

In addition, our numbers also indicate that convalescent individuals have a robust response against SARS-CoV-2, as has been also stated by other studies [20,21,22]. So, presumably, this response may play a fundamental role in the immune response against reinfection, as has been reported in other studies [23,24,25,26]. Moreover, convalescent individuals with a previous severe acute disease showed a higher cell response than those with milder forms. That increase in severe cases may be a result of either an exacerbated immune response during the disease, in combination with a greater initial viral inoculum (causing a severe disease and a larger adaptive response to the virus) [27,28]. In our study, convalescent individuals remained positive for both cellular and humoral responses, some of them even seven months after resolving the disease. It can therefore be assumed that after an acute SARS-CoV-2 episode, an individual is less susceptible to being infected due to the adaptive response generated. Despite this, reinfections have been reported to occur in convalescent individuals and are associated with lower risk of severe disease [13,29]. In fact, the risk of reinfection with older variants has been seen to be low, but increases with the emergence of new variants [30,31]. Information on variants affecting patients from our study can be of utility for understanding immune protection against reinfection. Despite not being this specific information recorded, the main circulating variants during the study period can be traceable according to our national Ministry of Health registries. Briefly, Alpha started to account for more than half of the circulating variants in January 2021. Next, Delta prevalence reached 5% in April 2021 and 95% in August 2021. Omicron represented more than 5% of cases for the first time in December 2021 [32].

When comparing the response between infection and vaccination, our study shows that an IFN-γ T-cell response is stronger in those patients who have undergone the disease than in those individuals who have been vaccinated. This should be further investigated, as the response has been described to be variable in vaccinated individuals and vaccines may not be equally effective against new SARS-CoV-2 variants [8]. The study published by Gazit S et al. showed that unvaccinated individuals had around a 13-fold increased risk to be infected with Delta variant than people with a past infection [33]. On the contrary, other studies have shown that vaccinated individuals had similar responses to convalescent ones, producing a cellular response at the same level as the humoral, even at an earlier stage. Moreover, in some vaccinated individuals a reduced IFN-γ T-cell response but an increased IL-2 T-cell response has been detected and may play a role in the long-term protection against infection [34,35]. Hence, for a better understanding of the T-cell response generated after vaccination and infection, other inflammatory cytokines should be studied. Spike has been seen to be the most immunogenic antigen of the three studied, although responses against NCP and membrane antigens must not be overlooked when comparing the immune response between natural infection and vaccination, as they also play a role in the protection against reinfection, generating a dominant response by cytotoxic T-cells, as reported in previous studies [20,36,37]. Finally, attending to the humoral response, overall results in our study indicate a moderate correlation between IgG and T-cell response levels for spike and NCP, as reported earlier [38]. However, we also observe that vaccination elicits a higher IgG response when compared to the IFN-γ T-cell one. Altogether, understanding which factors are responsible for these different responses is of great importance.

In addition to IFN-γ T-cell response, other inflammatory cytokines may be interesting to understand the immunopathology and the protection against reinfection. Looking into our results on IFN-γ, a high individual inter-variability was observed when monitoring COVID-19 patients and their outcomes, so it would be interesting to study other different cytokines and cell populations as possible prognosis markers. In line with this, sustained IL-6 and TNF-α production has been reported to be related to a low maturation of monocytes, also influencing the depletion of different cellular subsets including CD4+ T-cells [39]. These and other cytokines involved in the immunopathology of acute patients such as IL-1β and IL-10 could be fundamental for the discrimination of the outcome of the infection as they have been seen to be significantly higher in severe than in mild forms of the disease [15,40]. On the other hand, a strong and specific IL-2 response has been detected in COVID-19 recovered individuals [13,18,23], hence this cytokine’s role in immune protection should be considered. Further studies should aim to assess the secretion of other cytokines in the context of SARS-CoV-2 infection and vaccination as well as the involvement of different cell populations.

This study has several drawbacks that should be addressed. First, some groups such as acute COVID-19 or vaccinated individuals have a relatively small sample size; consequently, statistical strength can be reduced. However, even though this limitation, patients from these study groups are clinically and microbiologically well-characterized, being possible to investigate the immune response according to each clinical situation. Second, SARS-CoV-2 variants were not documented for the individuals that have been or were diagnosed with the virus, limiting the interpretation of the results depending on this factor. Despite not being able to classify each sample according to the virus variant causing the infection, this information could be traceable as periods of prevalence of the different variants are updated in epidemiological documents from our national Ministry of Health [32]. Third, vaccinated individuals were not followed-up, impeding the assessment of the response of each individual through time and the comparison of the response after first and second doses. In future studies the impact of third and coming fourth doses of the vaccine in T-cell response should be studied. Finally, classification according to severity of the COVID-19 disease followed in this study, as happens in other studies, did not completely fulfill with the classification recommended by WHO Working Group on the clinical characterization and management of COVID-19 [41], however it is rigorous and scientifically based to provide reliable results and conclusions.

Taking these facts into consideration, our findings show that measuring the T-cell responses is valuable to understand the picture of the immunity against SARS-CoV-2. We have provided data sustaining that spike, NCP, and membrane antigens from the virus can elicit the release of IFN-γ by specific T-cells, indicating that the last two antigens should not be overlooked in potential vaccine design and identification of the immune status. In addition, the IFN-γ T-cell response was low in the active phase of the disease, particularly in severe individuals. This response increased during convalescence, indicating that the adaptive T-cell response against the pathogen needs some time to be generated. According to the findings obtained for vaccinated individuals, our data suggest that the T-cell response is not always triggered after vaccination with an mRNA vaccine, however, it is compensated by the humoral immune response. Altogether, both types of responses are important against infection and towards protection and offer valuable information to understand the overall picture of the adaptive immunity against SARS-CoV-2.

## Figures and Tables

**Table 1:** Descriptive table from the patients included in the study.

Participants Variables		Controls (*n* = 128)				Acute (*n* = 42)						Convalescent (*n* = 60)					
	Unvaccinated (*n* = 80)		Vaccinated (*n* = 48)		Asymptomatic (*n* = 6)		Mild (*n* = 3)	Moderate (*n* = 16)	Severe (*n* = 17)			Mild (*n* = 22)			Moderate (*n* = 18)	Severe (*n* = 20)	
											Non-Hospitalized (*n* = 12)		Hospitalized (*n* = 10)				
**Age (years ± SD)**	39 ± 13.3		42.9 ± 13.9		37.8 ± 18.1		25.3 ± 3.5	56.4 ± 16.1	65.8 ± 14.1		42.9 ± 12.7		63.8 ± 9.7		58.1 ± 14.6	56.9 ± 11.3	
**Male N (%)**	24 (30)		9 (18.8)		4 (66.7)		0 (0)	13 (81.3)	13 (76.5)		3 (25)		6 (60)		10 (54.5)	15 (75)	
Pneumonia N (%) *	0 (0)		0 (0)		0 (0)		0 (0)	16 (100)	17 (100)		0 (0)		9 (90)		18 (100)	20 (100)	
*Unilobar*	0 (0)		0 (0)		0 (0)		0 (0)	1 (6.3)	0 (0)		0 (0)		2 (20)		2 (11.1)	0 (0)	
*Multilobar*	0 (0)		0 (0)		0 (0)		0 (0)	32 (93.7)	17 (100)		0 (0)		7 (70)		16 (88.9)	20 (100)	
ICU admission N (%) *	0 (0)		0 (0)		0 (0)		0 (0)	0 (0)	17 (100)		0 (0)		0 (0)		0 (0)	20 (100)	
Oxygen support N (%) *	0 (0)		0 (0)		0 (0)		0 (0)	16 (100)	17 (100)		0 (0)		5 (50)		18 (100)	20 (100)	
*Nasal prongs*	0 (0)		0 (0)		0 (0)		0 (0)	0 (0)	0 (0)		0 (0)		5 (50)		0 (0)	0 (0)	
*Non-invasive mechanical vent.*	0 (0)		0 (0)		0 (0)		0 (0)	16 (100)	0 (0)		0 (0)		0 (0)		18 (100)	0 (0)	
*Invasive mechanical vent.*	0 (0)		0 (0)		0 (0)		0 (0)	0 (0)	17 (100)		0 (0)		0 (0)		0 (0)	20 (100)	
**Vaccinated with 1st dose N (%)**	0 (0)		48 (100)		2 (33.3)		0 (0)	1 (6.3)	3 (17.6)		6 (50)		0 (0)		0 (0)	0 (0)	
**Vaccinated with 2nd dose N (%)**	0 (0)		40 (83.3)		0 (0)		0 (0)	0 (0)	0 (0)		4 (33.3)		0 (0)		0 (0)	0 (0)	
**Comorbidities N (%)**	2 (2.6)		9 (18.8)		0 (0)		0 (0)	14 (87.5)	12 (70.6)		3 (25)		6 (60)		12 (66.7)	12 (60)	
*Respiratory disorders* (asthma, OSAS, COPD)	0 (0)		2 (4.2)		0 (0)		0 (0)	9 (56.3)	1 (5.9)		0 (0)		4 (40)		1 (5.9)	0 (0)	
*Cardiovascular diseases* (AHT, ictus, atrial fibrillation)	1 (1.3)		2 (4.2)		0 (0)		0 (0)	7 (43.8)	10 (58.8)		1 (8.3)		3 (30)		8 (44.4)	9 (45)	
*Autoimmune disorders* (DM2, psoriasis, Jorgen, other)	1 (1.3)		4 (8.3)		0 (0)		0 (0)	4 (25)	6 (35.3)		1 (8.3)		1 (10)		4 (22.2)	5 (25)	
*Central nervous system disorders* (dementia, epilepsy, Parkinson)	0 (0)		1 (2.1)		0 (0)		0 (0)	2 (12.5)	4 (23.5)		0 (0)		0 (0)		1 (5.5)	1 (5)	
*Malignant neoplasies*	0 (0)		0 (0)		0 (0)		0 (0)	1 (6.3)	3 (17.6)		0 (0)		1 (10)		1 (5.5)	2 (10)	
*Obesity*	n/a		n/a		0 (0)		0 (0)	3 (18.8)	4 (23.5)		1 (8.3)		1 (10)		6 (33.3)	6 (30)	
**Immunosuppressive treatment N (%)**	2 (2.5)		4 (6.3)		0 (0)		1 (16.7)	4 (25)	1 (5.9)		1 (9)		1 (10)		3 (16.7)	2 (10)	
*Oral* (betamethasone, prednisone, NSAIDS)	1 (1.3)		1 (2.1)		0 (0)		1 (16.7)	1 (6.3)	1 (5.9)		0 (0)		0 (0)		0 (0)	1 (5)	
*Inhaled*	1 (1.3)		2 (4.2)		0 (0)		0 (0)	3 (18.8)	0 (0)		0 (0)		1 (10)		3 (16.7)	1 (5)	
*Topic*	0 (0)		2 (4.2)		0 (0)		0 (0)	0 (0)	0 (0)		1 (9)		0 (0)		0 (0)	0 (0)	
**Deaths N (%)**	0 (0)		0 (0)		0 (0)		0 (0)	0 (0)	5 (29.4)		0 (0)		0 (0)		0 (0)	0 (0)	

* In the convalescent group, these variables are referring to the characteristics of their acute COVID-19 episode. n/a = not available.

**Table 2:** Overall positivity results for the T-cell IFN-γ secretion against each antigen, including borderline results (%).

Groups	Spike (*n* = 252)	Nucleocapsid (*n* = 249)	Membrane (*n* = 249)	Any SARS-CoV-2 Antigen (*n* = 252)
**Overall (*n* = 259)**	118/252 (46.8)	77/249 (30.9)	73/249 (29.3)	130/252 (51.6)
**Controls (*n* = 128)**	27/125 (21.6)	2/125 (1.6)	3/125 (2.4)	27/125 (21.6)
Vaccinated (*n* = 48)	24/48 (50)	1/48 (2.1)	3/48 (6.3)	24/48 (50)
Unvaccinated (*n* = 80)	3/77 (3.9)	1/77 (1.3)	0/77 (0)	3/77 (3.9)
**Acute disease (*n* = 71)**	38/67 (56.7)	31/64 (48.4)	21/64 (32.8)	48/67 (71.6)
Asymptomatic (*n* = 6)	2/6 (33.3)	2/6 (33.3)	1/6 (16.7)	3/6 (50)
Mild (*n* = 3)	1/3 (33.3)	1/3 (33.3)	1/3 (33.3)	1/3 (33.3)
Moderate (*n* = 33)	22/32 (68.8)	14/32 (43.8)	8/32 (25)	24/32 (75)
Severe (*n* = 29)	13/26 (50)	13/23 (56.5)	10/23 (43.5)	20/26 (76.9)
**Convalescent (*n* = 60)**	53/60 (88.3)	44/60 (73.3)	49/60 (81.7)	55/60 (91.7)
Mild * (*n* = 22)	16/22 (72.7)	13/22 (59.1)	15/22 (69.6)	17/22 (78.3)
*Non-hospitalized (n = 12)*	6/12 (50)	6/12 (50)	5/12 (41.7)	7/12 (58.3)
*Hospitalized (n = 10)*	10/10 (100)	7/10 (70)	10/10 (100)	10/10 (100)
Moderate * (*n* = 18)	17/18 (94.5)	13/18 (72.2)	16/18 (88.9)	18/18 (100)
Severe * (*n* = 20)	20/20 (100)	18/20 (90)	18/20 (90)	20/20 (100)

Positivity percentages were calculated excluding the indeterminate results from the total number of samples tested for each group. Denominator of each ratio indicates the total *n* for that antigen and that group of individuals that had a valid result. * Severity considered during their acute COVID-19 episode.

**Table 3:** Overall positivity results obtained using antibody detection and T-cell IFN-γ production detection after spike and NCP stimulation including borderline results (%).

Groups	Antibody Response		T-Cell Response	
	IgG Spike (*n* = 155)	IgG NCP (*n* = 154)	Spike (*n* = 155)	NCP (*n* = 154)
**Overall (*n* = 159)**	86/155 (55.5)	55/154 (35.7)	63/155 (40.6)	32/154 (20.8)
**Controls (*n* = 94)**	33/92 (35.9)	4/92 (4.3)	17/92 (18.5)	1/92 (1.1)
Vaccinated (*n* = 30)	27/30 (90)	0/30 (0)	14/30 (46.7)	0/30 (0)
Unvaccinated (*n* = 64)	6/62 (9.7)	4/62 (6.5)	3/62 (4.8)	1/62 (1.6)
**Acute (*n* = 36)**	23/34 (67.6)	23/33 (69.7)	18/34 (52.9)	12/33 (38)
Asymptomatic (*n* = 6)	4/6 (66.7)	3/6 (50)	2/6 (33.3)	2/6 (33.3)
Mild (*n* = 3)	1/3 (33.3)	1/3 (33.3)	1/3 (33.3)	1/3 (33.3)
Moderate (*n* = 12)	8/12 (66.7)	9/12 (75)	9/12 (75)	4/12 (33.3)
Severe (*n* = 15)	10/13 (76.9)	10/12 (83.3)	6/13 (46.2)	5/12 (41.7)
**Convalescent (*n* = 29)**	29/29 (100)	28/29 (95.6)	28/29 (95.6)	20/29 (69)
Mild (*n* = 8)	8/8 (100)	7/8 (87.5)	8/8 (100)	6/8 (75)
Moderate (*n* = 10)	10/10 (100)	10/10 (100)	9/10 (90)	5/10 (50)
Severe (*n* = 11)	11/11 (100)	11/11 (100)	11/11 (100)	9/11 (81.8)

Positivity percentages were calculated excluding the indeterminate results from the total number of samples tested for each group. Denominator of each ratio indicates the total *n* for that antigen and that group of individuals that had a valid result.

## Data Availability

The data presented in this study are available on request from the corresponding author. The data are not publicly available due to privacy reasons.

## Supplementary Materials

The following supporting information can be downloaded at: https://www.mdpi.com/article/10.3390/jcm11175103/s1, Table S1. Descriptive table with information of the samples included in the study (time since diagnostic and vaccination, and lymphopenia at the moment of sampling); Table S2: Overall positive results for the T-cell IFN-γ secretion against high homology regions of endemic (human) coronaviruses included in the assay; Table S3: Overall positive results for SARS-CoV-2 antigens in samples from patients undergoing acute disease according to days after semi-intensive care admission including borderline results (%); Table S4: Comparison of the IFN-γ T-cell response with IgG response results. Overall comparison means positive for each of the antigens studied by both techniques (spike, NCP, and membrane for the IFN-γ T-cell studies, and spike and NCP for IgG detection); Figure S1: T-cell response (SFCs) follow-up in acute patients. Red lines indicate patients who died during the SARS-CoV-2 acute phase. Grey area shows borderline results; Figure S2: Correlation of the (a) IFN-γ T-cell response (SFCs; *n* = 54) and (b) IgG (*n* = 21) against spike in patients with acute disease and their lymphocyte concentration (lymphocyte/µL). The borderline area is shown in grey. On the left side of the discontinuous vertical line (<1200 lymphos/µL) lymphopenia is considered. Correlation was calculated using the two-tailed non-parametric Spearman test; Figure S3. Number of SFCs after stimulation with NCP (a), and membrane (b) in the different study groups. Horizontal lines represent medians. Grey area shows borderline results. Differences between conditions were calculated using the two-tailed Mann-Whitney U test. *p* is considered significant when <0.05 (** <0.01, *** <0.001, and **** <0.0001).
