# Supplementary material for: Measuring T-Cell Responses against SARS-CoV-2 Is of Utility for Disease and Vaccination Management
Source: J Clin Med. 2022 Aug 30;11(17):5103. doi: 10.3390/jcm11175103 (PMC9457376; doi:10.3390/jcm11175103)
Supplement: Supplementary file 1 [file jcm-11-05103-s001.zip › jcm-1859629-supplementary.pdf]

Table S1. Descriptive table with information of the samples included in the study (time since diagnostic and vaccination, and lymphopenia at the moment of sampling).

Samples Variables		Controls (n = 128)		Acute (n = 71)				Convalescent (n = 60)			
								Mild (n = 22)			
		Unvaccinated (n = 80)	Vaccinated (n = 48)	Asymptomatic (n = 6)	Mild (n = 3)	Moderate (n = 33)	Severe (n = 29)	Non-Hospitalized (n = 12)	Hospitalized (n = 10)	Moderate (n = 18)	Severe (n = 20)
**Time since diagnosis (days± SD)**		-	-	10.8±6	0±0	9.1±6.6	13.2±10.8	45.6±55.8*	78±57	94.9±42.6	117.6±33.1
**Time since 1st vaccine dose (days± SD)**		-	52.8±51.9	9.5±4.9	-	11±0	16±3.7	129.8±135.2	-	-	-
**Time since 2nd vaccine dose (days± SD)**		-	32.2±18	-	-	-	-	161±137.7	-	-	-
**Lymphopenia at sampling N (%)****	*Yes*	0 (0)	0 (0)	0 (0)	0 (0)	13 (39.4)	19 (65.5)	0 (0)	1 (10)	2 (11.1)	0 (0)
	No	12 (15)	3 (6.3)	1 (16.7)	0 (0)	18 (54.5)	9 (31)	1 (8.3)	8 (80)	16 (88.9)	20 (100)
	Not reported	68 (85)	45 (93.7)	5 (83.3)	3 (100)	2 (6.1)	1 (3.5)	11(91.7)	1 (10)	0 (0)	0 (0)

*Days since diagnosis was not registered for one of the participants. **Having a concentration of lymphocytes below 1200 cells/uL was considered as lymphopenia.

Table S2. Overall positive results for T-cell IFN-γ secretion against high homology regions of endemic (human) coronaviruses included in the assay.

**Groups**	High homology regions with common CoV (*n* = 251)
**Overall (*n* = 259)**	65/251 (25.9)
**Controls (*n* = 128)**	17/125 (13.6)
Vaccinated (*n* = 48)	14/48 (29.2)
Unvaccinated (*n* = 80)	3/77 (3.9)
**Acute disease (*n* = 71)**	21/66 (31.8)
Asymptomatic (*n* = 6)	2/6 (33.3)
Mild (*n* = 3)	1/3 (33.3)
Moderate (*n* = 33)	11/32 (34.4)
Severe (*n* = 29)	7/25 (28)
**Convalescent (*n* = 60)**	27/60 (45)
Mild* (*n* = 22)	18 (47.8)
Non-hospitalized (n = 12)	6/12 (50)
Hospitalized (n = 10)	4/10 (40)
Moderate* (*n* = 18)	8/18 (45.5)
Severe* (*n* = 20)	9/20 (45)

Positivity percentages were calculated excluding the indeterminate results from the total number of samples tested for each group. Denominator of each ratio indicates the total n for that antigen and that group of individuals that had a valid result. * Severity considered during their acute COVID-19 episode.

Table S3. Overall positive results for SARS-CoV-2 antigens in samples from patients undergoing acute disease according to days after semi-intensive care admission including borderline results (%).

**Days after respiratory semi-intensive care admission**	P1 (Spike)	P3 (NCP)	P4 (Membrane)	Any reactive SARS-CoV-2 panel
**Overall (*n* = 48)**	26 (54.2)	22 (45.8)	14 (29.2)	34 (70.8)
Day 0 (*n* = 13)	3 (23.1)	6 (46.2)	2 (15.4)	7 (53.8)
Day 2 (*n* = 18)	9 (50)	9 (50)	4 (16.7)	13 (72.2)
Day 7 (*n* = 12)	9 (75)	4 (33.3)	4 (33.3)	9 (75)
Discharge (*n* = 2)*	2 (100)	1 (50)	2 (100)	2 (100)
Day 28 (*n* = 3)	3 (100)	2 (66.7)	2 (66.7)	3 (100)

*Discharge samples were taken at day 10 and 13 after Respiratory semi-intensive care admission.

Table S4. Comparison of IFN-γ T-cell response with IgG response results. Overall comparison means positive for each of the antigens studied by both techniques (spike, NCP and membrane for IFN-γ T-cell studies, and spike and NCP for IgG detection).

		**T-cell response spike**		**T-cell response NCP**		**T-cell response overall**	
**Unvaccinated (*n* = 62)**		**Pos**	**Neg**	**Pos**	**Neg**	**Pos**	**Neg**
IgG response	**Pos**	1	5	0	3	1	6
	**Neg**	2	54	1	58	2	53
**Vaccinated (*n* = 30)**							
IgG response	**Pos**	14	14	0	0	14	14
	**Neg**	0	2	0	30	0	2
**Acute patients (*n* = 34*)**							
IgG response	**Pos**	13	9	8	13	18	7
	**Neg**	5	7	4	8	4	5
**Convalescent (*n* = 29)**							
IgG response	**Pos**	28	1	19	9	29	0
	**Neg**	0	0	1	0	0	0

**n* = 33 for NCP

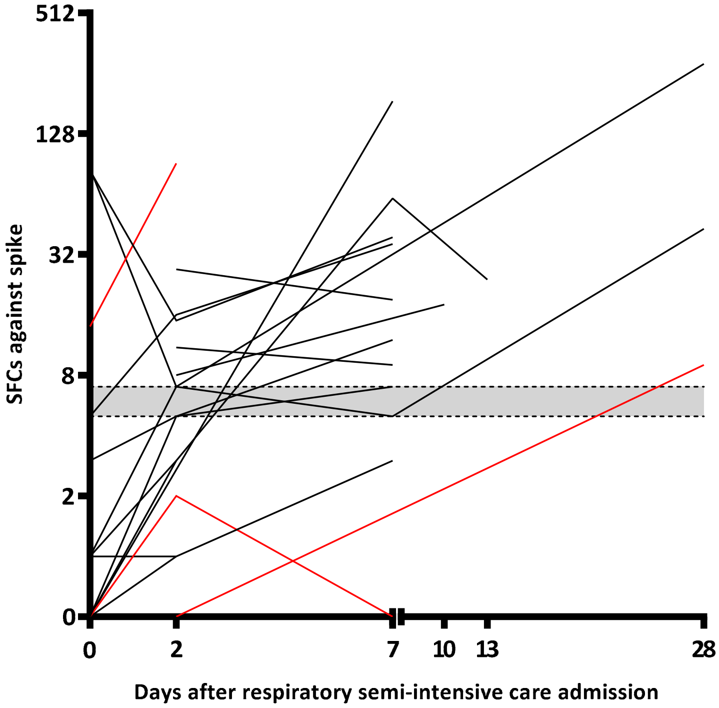

Figure S1. T-cell response (SFCs) follow-up in acute patients. Red lines indicate patients who died during the SARS-CoV-2 acute phase. Grey area shows borderline results.

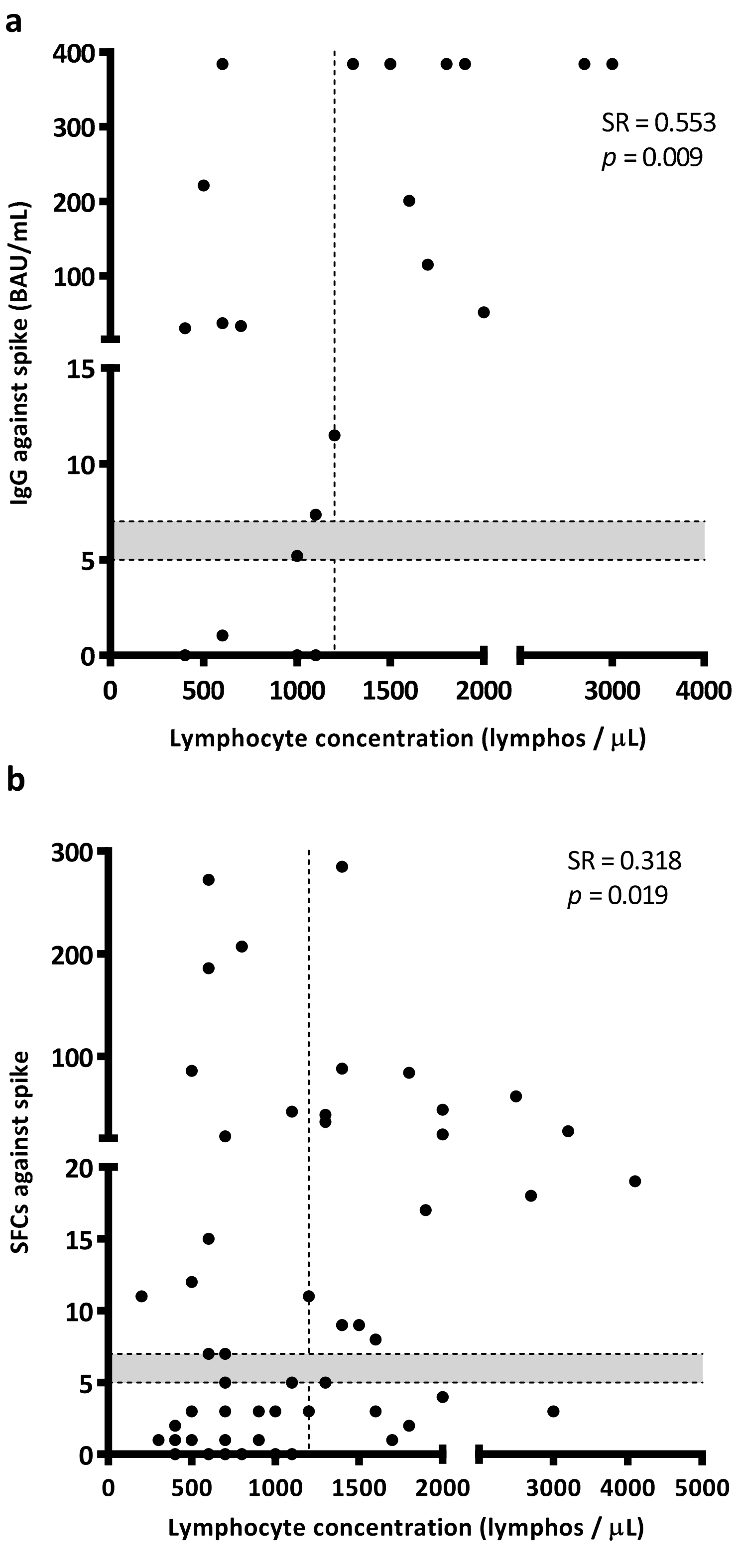

Figure S2. Correlation of the a) IFN-γ T-cell response (SFCs; n = 54) and b) IgG (n = 21) against spike in patients with acute disease and their lymphocyte concentration (lymphocyte/µl). The borderline area is shown in grey. On the left side of the discontinuous vertical line (<1200 lymphos/µL) lymphopenia is considered. Correlation was calculated using the two-tailed non-parametric Spearman test.

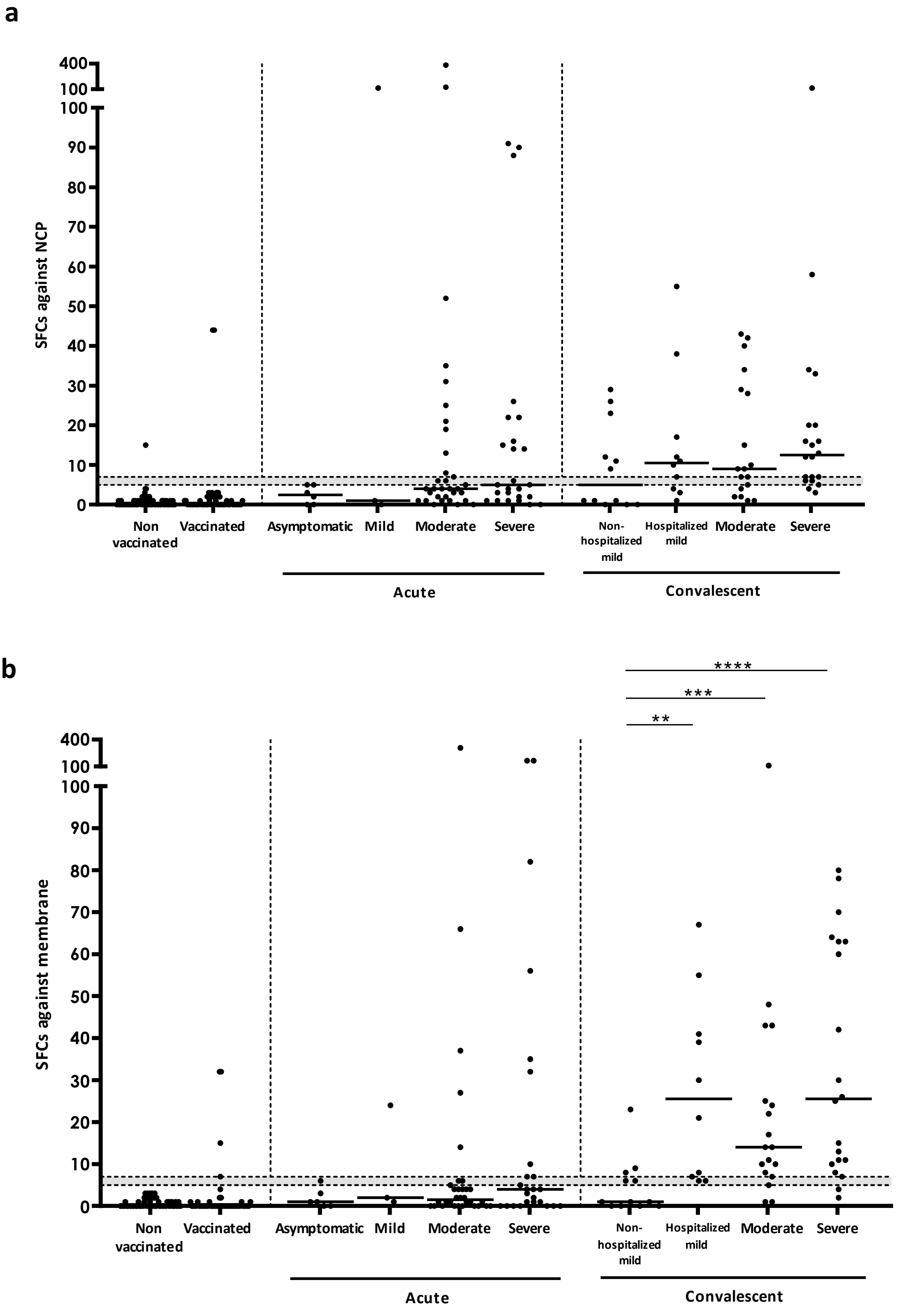

Figure S3. Number of SFCs after stimulation with NCP (a), and membrane (b) in the different study groups. Horizontal lines represent medians. Grey area shows borderline results. Differences between conditions were calculated using the two-tailed Mann-Whitney U test. p is considered significant when <0.05 (** <0.01, *** <0.001, and ****<0.0001).
